# Longitudinal changes in resting state fMRI brain self-similarity of asymptomatic high school American football athletes

**DOI:** 10.1038/s41598-024-51688-2

**Published:** 2024-01-19

**Authors:** Bradley Fitzgerald, Sumra Bari, Nicole Vike, Taylor A. Lee, Roy J. Lycke, Joshua D. Auger, Larry J. Leverenz, Eric Nauman, Joaquín Goñi, Thomas M. Talavage

**Affiliations:** 1 Elmore Family School of Electrical and Computer Engineering, Purdue University, West Lafayette, IN USA; 2 Department of Computer Science, University of Cincinnati, Cincinnati, OH USA; 3 Department of Biomedical Engineering, University of Cincinnati, Cincinnati, OH USA; 4 Department of Basic Medical Sciences, Purdue University, West Lafayette, IN USA; 5 School of Mechanical Engineering, Purdue University, West Lafayette, IN USA; 6 Department of Electrical and Computer Engineering, Rice University, Houston, TX USA; 7 Department of Health and Kinesiology, Purdue University, West Lafayette, IN USA; 8 Weldon School of Biomedical Engineering, Purdue University, West Lafayette, IN USA; 9 School of Industrial Engineering, Purdue University, West Lafayette, IN USA; 10 Purdue Institute for Integrative Neuroscience, Purdue University, West Lafayette, IN USA

**Keywords:** Brain injuries, Biomedical engineering, Neuroscience

## Abstract

American football has become the focus of numerous studies highlighting a growing concern that cumulative exposure to repetitive, sports-related head acceleration events (HAEs) may have negative consequences for brain health, even in the absence of a diagnosed concussion. In this longitudinal study, brain functional connectivity was analyzed in a cohort of high school American football athletes over a single play season and compared against participants in non-collision high school sports. Football athletes underwent four resting-state functional magnetic resonance imaging sessions: once before (pre-season), twice during (in-season), and once 34–80 days after the contact activities play season ended (post-season). For each imaging session, functional connectomes (FCs) were computed for each athlete and compared across sessions using a metric reflecting the (self) similarity between two FCs. HAEs were monitored during all practices and games throughout the season using head-mounted sensors. Relative to the pre-season scan session, football athletes exhibited decreased FC self-similarity at the later in-season session, with apparent recovery of self-similarity by the time of the post-season session. In addition, both within and post-season self-similarity was correlated with cumulative exposure to head acceleration events. These results suggest that repetitive exposure to HAEs produces alterations in functional brain connectivity and highlight the necessity of collision-free recovery periods for football athletes.

## Introduction

Recently, American football has become the focus of numerous studies^1–7^ elucidating potential health risks associated with exposure to repetitive head acceleration events (HAEs). Exposure to HAEs that do not result in a clinical concussion diagnosis have been demonstrated to cause various neurological alterations in athletes^1,8–13^. Given high school football players typically experience over 500 HAEs per season, with some athletes sustaining over 2000^14–17^, it is critical that the relationship between HAEs and brain physiology is well-understood. Such understanding is especially important to promote the health of youth athletes, as younger players have both an increased risk of experiencing traumatic brain injury and an increased risk of developing long-term negative cumulative consequences following HAE exposure^18–21^.

Brain physiology in collision sports athletes has widely been studied using a variety of magnetic resonance imaging (MRI) modalities^7,10,17,22^. For instance, structural MRI methods (e.g., T1 imaging) have demonstrated alterations in brain tissue volumes in youth collision athletes^8^. Diffusion-weighted imaging techniques have shown that repetitive exposure to HAEs can result in changes in white matter tracts^3,15,23,24^. Magnetic resonance spectroscopy techniques have found a link between altered neurometabolic concentrations and exposure to HAEs^13,25,26^. MRI-derived measures of cerebrovascular reactivity changes have also been shown to be associated with cumulative HAEs in athletes^6,12^.

Of particular relevance for the current study are functional MRI (fMRI) techniques, which have demonstrated potential in quantifying physiological brain alterations due to HAEs^17,27^. fMRI records voxel-wise blood-oxygen-level-dependent (BOLD) time series, from which low-frequency signal oscillations are analyzed to assess functional connectivity between brain regions^28^. Previous studies have demonstrated a link between functional connectivity changes and sports-related concussions^29,30^. Further, repetitive sports-related exposure to HAEs has been shown to be associated with brain alterations in both task-based functional activity^17,22,31^ and in resting state functional connectivity^1,11,32–34^. Analysis of brain functional connectivity has led to the development of functional connectomes (FCs), which are square, symmetric matrices of pairwise correlations between average BOLD signals derived from brain regions of interest^35^. Previously, it has been shown that individual FCs are identifiable from within a cohort of participants, as repeated FC acquisitions tend to be more highly correlated with oneself than with others^36–38^. Given that healthy individuals tend to exhibit similar FC patterns across multiple imaging sessions, we aimed in the current study to quantify intra- (self-) and inter-individual similarity of repeated FC acquisitions in order to analyze the degree of functional brain changes occurring within young athletes exposed to HAEs. Our hypothesis was that collision-sport athletes who are exposed to HAEs would exhibit more pronounced longitudinal changes in self-similarity of FCs than would control athletes who do not participate in collision-based activities.

Even amidst growing effort to understand brain physiology in relation to sports-related HAEs, the current literature is still lacking in prospective studies with collection of data at multiple time points. A more robust understanding of the influence of collision sports participation on brain health requires analysis of athletes at multiple time points before, during, and after the play season. Further, study of asymptomatic athletes presents the unique challenge of defining which biometrics represent a harmful alteration to the athletes’ brain physiology; to date, such biometrics have not been clearly defined. In response to these gaps, the current effort prospectively monitored a cohort of high school American football athletes to assess longitudinal changes in functional connectivity throughout the competition season.

In this study, athletes underwent four resting state fMRI scan sessions over the course of a play season, with FCs computed for each scan session. For each participant, FCs from each scan session were compared with a pre-season (scan session before the start of the season’s contact practices) FC to evaluate the degree of self-similarity of the FCs throughout the play season, forming a method for identifying concerning levels of functional alterations in players. This analysis was completed on a whole-brain level and for seven sub-levels corresponding to seven functional brain networks. In addition, the measure of FC self-similarity throughout the season was assessed for correlation with the number and magnitude of HAEs experienced by the athletes.

## Methods

### Cohort information

All study procedures were compliant with the ethical principles of the Belmont Report and the Declaration of Helsinki and received Institutional Review Board (Purdue University) approval prior to data collection. Recruitment of athletes was conducted through a presentation of the study aims at the time of pre-participation meeting between players’ parents, coaching staff, and school administrators. Athletes were informed that participation in the study was voluntary and would not impact their academic or athletic status. For each study participant, if the player was under 18 years of age, then informed consent was obtained from their legal guardian, along with assent of the player. If the player was at least 18 years old, the player provided informed consent.

72 male athletes participated in this study. *Football Athletes (FBA):* 58 of these athletes were active participants in American football at the high school varsity or junior varsity level. Of the FBA cohort, 16 participants were excluded from analysis after imaging data quality checks (described in subsequent sections). *Non-Collision Athletes (NCA):* The remaining 14 athletes were active participants in typically collision-free sports at the high school varsity or junior varsity level and were imaged as controls. Demographics information (including age, race, and NCA sports participation) of all participants included in analysis is displayed in Table 1. No significant difference existed in the ages of the FBA and NCA groups (Wilcoxon rank sum test, *p* = 0.17). Demographic measures were not used in any analyses and are provided only for informational purposes.

**Table 1:** Demographics of participants included in analysis (i.e., with full set of valid imaging data).

		FBA (n = 42)	NCA (n = 14)
Age (years)	Mean ± StDev Median	16.7 ± 1.0 17	16.2 ± 1.1 16.5
	[Min, Max]	[15, 18]	[14, 18]
Racial/Ethnic Categories	White	24	13
	Black or African American	7	0
	Hispanic or Latino	5	0
	Asian	2	1
	More than one	3	0
	Other (unspecified)	1	0
NCA Sport (some participated in multiple sports)	Basketball	–	1
	Cross Country	–	5
	Track and Field	–	8
	Swimming	–	4
	Golf	–	1

### Imaging timeline

**Figure 1:**
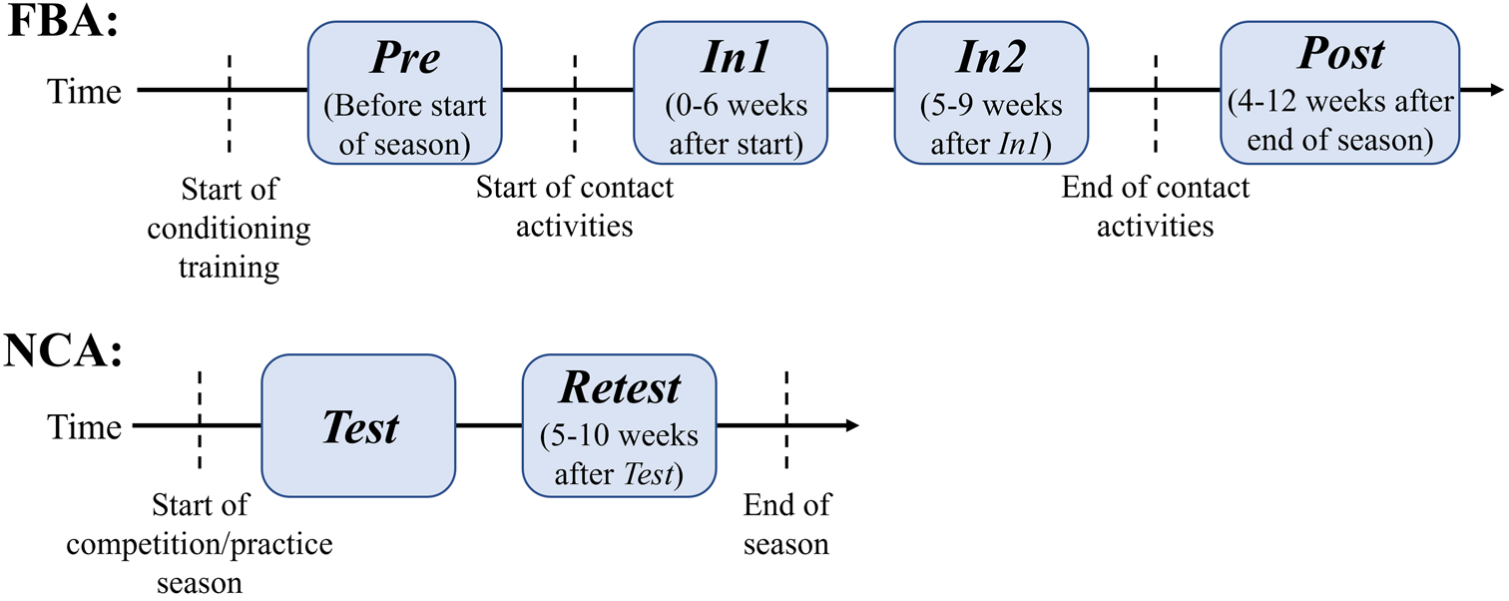
Imaging timeline for football athletes (FBA) and non-collision sports athletes (NCA). FBA underwent four imaging sessions (*Pre, In1, In2*, and *Post*) over the course of a single football play season. The *Pre* session occurred after the start of physical conditioning training but before the start of contact activities. The *In1* and *In2* scan sessions occurred during the period of contact activities, and the *Post* scan session occurred after contact activities had ceased. Since NCA were not expected to experience sports-related head acceleration events (HAEs), NCA were imaged twice (*Test, Retest*) during the competition season for their respective sports.

FBA underwent four imaging sessions spread over the course of a football competition season (Fig. 1). The first imaging session (*Pre*) was conducted prior to the start of the season’s “contact” practices, though following the point at which athletes had started regular physical fitness conditioning. The second session (*In1*) was conducted within the first half of the competition season, after the start of contact activities. The third session (*In2*) was conducted during the second half of the competition season (5–9 weeks after *In1*). The fourth session (*Post*) was conducted 4–12 weeks after contact activities were ended. NCA underwent two imaging sessions (*Test* and *Retest*), 5–10 weeks apart, during the active practice and/or competition season for their respective sports.

### MRI data collection

All MRI data were collected using a General Electric 3 T Signa HDx (Waukesha, WI) scanner with a 16-channel brain array (Nova Medical; Wilmington, MA), located at the Purdue University MRI Facility (West Lafayette, IN). For each imaging session, a T1-weighted structural brain image (fast spoiled gradient-recalled echo; TR/TE = 5.7/1.976 ms; flip angle = 73°; 1 mm isotropic) and resting state fMRI (rs-fMRI) data (gradient-echo echo-planar; scan length 9 min 48 s; 294 volumes acquired; TR/TE = 2000/26 ms; flip angle = 35; FOV = 20 cm; 64 × 64 acquisition) were collected. Foam padding was used to restrain head motion during scanning. Participants were asked to remain awake and keep their eyes open during fMRI acquisitions.

### HAE monitoring

During contact activities, football athletes wore a head-mounted xPatch sensor (X2 Biosystems, Inc, Seattle, WA) which recorded peak linear acceleration (PLA) of HAEs. Head-mounted sensors offer greater accuracy than helmet-mounted sensors and fewer safety risks than mouth guard-based sensors^10^. Use of the xPatch sensor was motivated by a previous study which found that the xPatch produced the lowest PLA measurement error levels when compared with several other commercially available sensor packages^39^. Sensors were positioned, using an adhesive patch, on the skin over the right mastoid process, immediately behind the pinna (as depicted in previous work^40^). Only acceleration events with PLA exceeding 20 g were considered for this study^40–42^. No HAE monitoring was conducted on NCA, as no events exceeding 20 g were anticipated for this population.

### Data preprocessing

Data preprocessing and analysis were implemented using in-house MATLAB (https://mathworks.com/) code. All T1 structural images and rs-fMRI data were preprocessed using AFNI^43^ and FSL^44,45^ in accordance with steps described by Bari et al.^37^. First, T1 structural images underwent denoising and bias-correction (FSL *fsl_anat*) using denoising filters^46–48^. Denoised structural images then underwent intensity normalization (AFNI *3dUnifize*) and were segmented into gray matter (GM), white matter (WM), and cerebrospinal fluid (CSF) tissue regions (FSL *fast*).

Preprocessing of rs-fMRI data was conducted in the native individual space. To mitigate spin history effects, the first four acquired fMRI volumes were removed. The remaining BOLD timeseries then underwent outlier detection (AFNI *3dToutcount*), where volumes were censored if more than 10% of the voxels in the volume were identified as outliers (a total of 294 volumes were originally acquired). The timeseries then underwent despiking (AFNI *3dDespike*), slice timing correction (AFNI *3dTshift*), and volume registration (AFNI *3dvolreg*) to the volume with fewest outlier voxels. The resulting timeseries was then aligned with the T1 structural image (AFNI *align_epi_anat.py*). Voxel-wise spatial smoothing, with a 4 mm full-width-at-half-maximum isotropic Gaussian kernel, was separately applied within the GM, WM, and CSF masks (AFNI *3dBlurinMask*). For each voxel, the voxel timeseries was divided by the mean of the voxel timeseries, multiplied by 100, and a maximum cutoff value of 200 applied. In addition to outlier volume censorship, volumes were also censored if the Euclidean norm of motion derivatives (computed during volume registration) exceeded 0.4^49^. For any censored volume, both that volume, the preceding volume, and the following volume were removed. If any fMRI time series had over one-third of volumes censored, the associated individual was removed from the study. Sixteen FBA had at least one scan session fMRI dataset fail these quality checks and were thus removed, leaving 42 football participants included in this study.

fMRI timeseries were detrended (AFNI *3dDeconvolve*) using the following regressors: (1) very low frequency fluctuations, computed using a 0.002–0.01 Hz bandpass filter (AFNI *1dBport*); (2) 12 motion parameters (computed during motion correction) including three linear translation parameters, three rotation parameters, and the first derivatives of each^50,51^; and (3) voxel-wise average WM time series computed within a 40-mm local neighborhood (AFNI *3dTproject*)^52^.

GM voxels were parcellated into 278 regions of interest (ROIs) based on the GM atlas from Shen et al.^53^. Application of this parcellation to individual participants was completed by registration of the atlas to each participant’s T1 structural image using non-linear registration (AFNI *auto_warp.py* and *3dAlineate*). fMRI data from some participants did not cover the full cerebellum region, so the (30) ROIs corresponding to the cerebellum were removed, leaving 248 GM ROIs. Symmetric 248 × 248 functional connectivity matrices were computed for each scan session, where the entry at location *(i, j)* is computed as the Pearson’s linear correlation coefficient (MATLAB *corr*) between averaged fMRI time series of ROIs *i* and *j*. We refer to these matrices as functional connectomes (FCs).

### Data analysis

#### Whole-brain FBA self-similarity distributions

FC similarity between two scan sessions was evaluated using the metric *self-similarity* (*I*_*self*_)^36,37^. Since a FC is a square and symmetric matrix, *I*_*self*_ between two scan sessions of an individual was computed as the Pearson’s correlation coefficient between the two vectorized upper triangular portions of the FC matrices. *I*_*self*_ reflects the similarity between two scan sessions of an individual. For FBA, *I*_*self*_ was computed comparing the following scan session pairs: *Pre-In1*, *Pre-In2*, *Pre-Post* (see Fig. 1 for session details).

**Figure 2:**
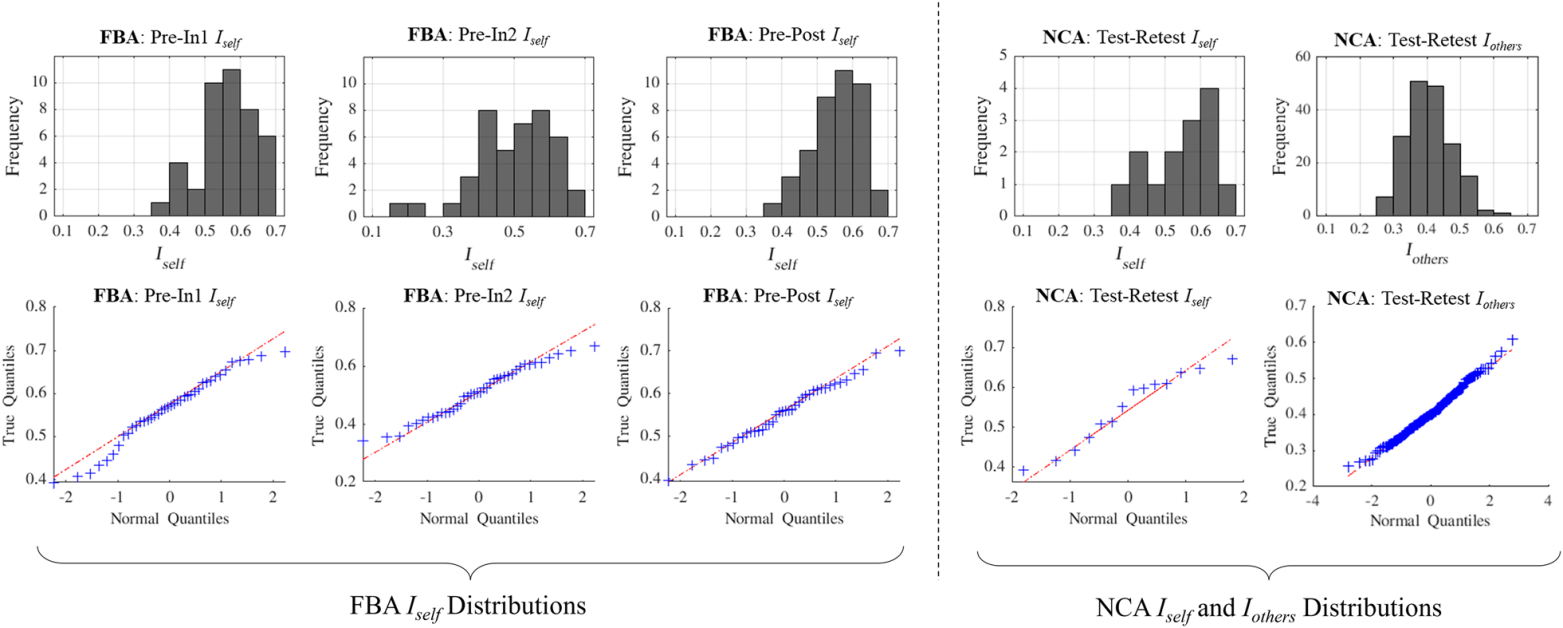
(Top) *I*_*self*_ and *I*_*others*_ histograms presented as a function of population and session comparison. (Bottom) Quantile–quantile (Q-Q) plots for assessing normality of the above distributions. Football athletes' *Pre-In2 I*_*self*_ measures were found to reject the null hypothesis of a normal distribution. *I*_*self*_ measures for all other computed session comparisons, including non-collision athletes (NCA: *Test–Retest*) and football athletes (FBA: *Pre-In1*, *Pre-Post*), were found to be normal. Additionally, the NCA *I*_*others*_ distribution was deemed normal.

Analyses were conducted to evaluate the normality of FBA *I*_*self*_ distributions and determine the proper statistical tests for further analyses. The distributions of *I*_*self*_ measurements for each FBA scan pairing (*Pre-In1, Pre-In2, Pre-Post*) were tested for normality using the Shapiro–Wilks normality test. Normality was visually inspected using Q-Q plots for each distribution (Fig. 2). The three FBA *I*_*self*_ distributions were also tested for equal variance using a Bartlett test. Only the *Pre-In2 I*_*self*_ distribution did not pass the normality test and the Bartlett test returned the conclusion that the three *I*_*self*_ distributions did not have equal variance, leading us to use non-parametric statistical tests in the remaining analyses directly using FBA *I*_*self*_ values. These three FBA *I*_*self*_ distributions were pairwise-tested for differences in medians using Wilcoxon signed rank tests (MATLAB *signrank*) with the Benjamini–Hochberg false discovery rate (FDR) correction procedure^54^ applied for multiple (n = 3, as three pairings of *I*_*self*_ distributions were assessed) comparisons (i.e., *p*_*FDR*_ < 0.05 indicates significance). We note that within this paper, all subsequent uses of the term FDR correction refer to this Benjamini–Hochberg FDR correction procedure (implemented using MATLAB *fdr_bh*^55^).

#### NCA ***I***_***self***_ and ***I***_***others***_ distributions

For control NCA, *I*_*self*_ was only computed comparing the two sessions *Test* and *Retest*. Such *I*_*self*_ for NCA reflects changes in the FC of athletes that are not due to sports-related exposure to HAEs. The three FBA *I*_*self*_ distributions (*Pre-In1, Pre-In2,* and *Pre-Post*) were each tested for significant difference against the NCA *Test–Retest I*_*self*_ distribution using the Wilcoxon rank sum test (MATLAB *ranksum*) with FDR correction for multiple (n = 3) comparisons.

In addition, it is of interest to understand the “floor” level of *I*_*self*_ that should be anticipated in the absence of HAE exposure. This is particularly important when exploring whether the *I*_*self*_ measure changes in a meaningful manner as a consequence of HAE exposure. To this end, we aimed to quantify the level at which *I*_*self*_ measurements may be deemed sufficiently low that the participant may be considered to be as distant from themselves (as assessed at baseline) as if they were an altogether separate individual. Similar to *I*_*self*_ measurements, analogous correlation comparisons can be made on full connectivity profiles between different subjects. The set of pairwise comparisons across *Test–Retest* FCs from different subjects form what we denominate here as the *I*_*others*_ distribution (note that the mean of such a distribution has been defined simply as *I*_*others*_ in an earlier study^36^). Here, we computed the *I*_*others*_ distribution as the set of pairwise FC (Pearson’s) correlation coefficients between all individuals in the NCA population to serve as a baseline distribution for expected FC similarity across individuals. Note that for a given pairing (i.e., Subject *i* and Subject *j*) of NCA participants (n = 14), there are two *I*_*others*_ values, first comparing Subject *i*’s *Test* with Subject *j’s Retest* and second comparing Subject *i*'s *Retest* with Subject *j*’s *Test*. All such pairwise correlation coefficients were computed resulting in a distribution for *I*_*others*_ comprising 182 measurements.

The NCA *Test–Retest I*_*self*_ and *I*_*others*_ distributions were both tested for normality using the Shapiro–Wilks normality test and were assessed using Q–Q plots (Fig. 2). Neither distribution resulted in rejection of the null hypothesis, so the distributions were both deemed normal. 95% confidence intervals were computed for the NCA *Test–Retest I*_*self*_ and *I*_*others*_ distributions as follows: for a given distribution, the estimated mean ($\mu$) and standard deviation ($\sigma$) of the distribution were computed, and the 95% interval defined as [$\mu \pm 1.96\sigma$]. For each set (*Pre-In1, Pre-In2,* and *Pre-Post*) of FBA *I*_*self*_ values, the total number of individual *I*_*self*_ values which fell (1) within the NCA *Test–Retest I*_*self*_ confidence interval and (2) below the upper limit of the NCA *I*_*others*_ confidence intervals was computed. A one-sided binomial test was used to test whether a significantly greater proportion of FBA athletes had *I*_*self*_ lower than the upper limit of the NCA *Test–Retest I*_*others*_ 95% confidence interval as compared with NCA *I*_*self*_. This test was conducted for the FBA *Pre-In1, Pre-In2,* and *Pre-Post I*_*self*_ distributions.

#### ***I***_***self***_ analysis within resting state networks

*I*_*self*_ is a similarity score on FCs obtained from the same individual at different times. Furthermore, *I*_*self*_ can be computed not only on whole-brain FC profiles, but also focused on specific functional networks (e.g., as proposed by Yeo et al.^56^) by considering only ROIs corresponding to a specific network when computing *I*_*self*_. For each of the seven functional networks proposed by Yeo et al.^56^, network-specific *I*_*self*_ values were computed for each participant. Specifically, network-specific *I*_*self*_ values were computed for FBA *Pre-In1*, *Pre-In2*, and *Pre-Post* session pairings and for the NCA *Test–Retest* pairing. Network-specific, pairwise comparisons were conducted between the three FBA *I*_*self*_ distributions (*Pre-In1, Pre-In2*, and *Pre-Post*) for each of the seven functional networks using the Wilcoxon signed rank test (MATLAB *signrank*) with FDR correction for multiple comparisons (n = 3, as three pairings of *I*_*self*_ distributions were assessed). In addition, each of the FBA *I*_*self*_ distributions were tested against the NCA *Test–Retest I*_*self*_ distribution for each network using the Wilcoxon rank sum test (MATLAB *ranksum*) with FDR correction for multiple comparisons (n = 3).

#### Assessing the effect of accumulated HAEs on ***I***_***self***_

To investigate association between functional connectivity changes and the HAEs experienced by FBA, several correlation analyses were conducted. Three HAE metrics were calculated for each scan session (*In1*, *In2,* and *Post*): total number of HAEs, cumulative PLA^12^, and average PLA. The total number of HAEs (*nHAEs*_*Th,i,j*_) experienced by the *i-th* athlete prior to the *j-th* scan session was computed by counting each of the *N*_*i,j*_ HAEs exceeding a chosen threshold (*Th)*:

$$nHA{Es}_{Th,i,j}= \sum_{k=1}^{{N}_{i,j}}u(PLA{}_{k,i}-Th)$$

where

$$u\left(x\right)= \left\{\begin{array}{l}1 \; \; \; \text{if} \; \; \; x>0\\ 0 \; \; \; \text{if} \; \; \; x\le 0\end{array}\right.$$

The cumulative PLA (*cPLA*_*Th,i,j*_) of HAEs experienced prior to session *j* was computed by summing the PLA of each of the *N*_*i,j*_ HAEs which exceeded a chosen threshold^25^:

$${cPLA}_{Th,i,j}= \sum_{k=1}^{{N}_{i,j}}{PLA}_{k,i}\cdot u(PLA{}_{k,i}-Th)$$

Finally, the average PLA (*aPLA*_*Th,i,j*_) of HAEs exceeding threshold *Th* and experienced prior to session *j* was computed as:

$$aPL{A}_{Th,i,j}= \frac{cPL{A}_{Th,i,j}}{nHA{E}_{Th,i,j}}$$

These three metrics were computed for the thresholds $Th\in \{20 g, 30 g, \dots , 70 g\}$.

The relationship between FBA *Pre-In1 I*_*self*_, *Pre-In2 I*_*self*_, and HAE metrics was assessed using Spearman’s correlation coefficient for each threshold value. Since the FBA *Pre-In2 I*_*self*_ distribution did not pass the normality test (as described previously), a non-parametric method was chosen for analysis with HAE metrics. Analysis of the comparison between *Pre-In1 I*_*self*_ values and *nHAE*_*In1*_ at the PLA thresholds revealed several datapoints that were outliers based on Cook’s distance (an outlier is defined as a datapoint with a Cook’s distance greater than three times the average Cook’s distance over all datapoints). Spearman’s correlation coefficient $\rho$ was computed (MATLAB *corr* with Spearman option applied) assessing FBA *Pre-In1 I*_*self*_ with *nHAEs*_*In1*_*, cPLA*_*In1*_*,* and *aPLA*_*In1*_. We determined a prior hypothesis that higher HAE metrics would correspond with lower *I*_*self*_ values, so the significance of these correlations were assessed using a left-tailed test (i.e., with null hypothesis $\rho \ge 0$ and alternative hypothesis $\rho <0$). Given (1) that the primary goal of this analysis was to determine which PLA threshold maximized the correlation of HAE metrics with *I*_*self*_ and (2) the direct interdependence (and thus high correlation) between HAE metrics across consecutive PLA thresholds, no multiple comparisons correction was applied for this analysis.

To ensure that this correlation assessment was robust, for any pairing which produced significant correlation, 10,000 iterations of bootstrapped random sampling (with replacement) were conducted. With each iteration, Spearman’s $\rho$ was computed, yielding 10,000 estimates of the coefficient. For a given correlation assessment at each PLA threshold, the HAE metric (*nHAEs, cPLA,* or *aPLA*) was considered to have a significant effect on *Pre-In1 I*_*self*_ if at least 95% of bootstrapped iterations produced $\rho$ less than 0. Similarly, this correlation assessment was conducted assessing *Pre-In2 I*_*self*_ with *In2* HAE metrics and comparing *Pre-Post I*_*self*_ with *Post* HAE metrics.

## Results

In summary, four sets of analyses were conducted: (1) FBA *I*_*self*_ distributions (*Pre-In1, Pre-In2*, and *Pre-Post*) underwent pairwise comparisons to evaluate FC changes over the course of the play season; (2) FBA *I*_*self*_ distributions were compared against control NCA *I*_*self*_ and *I*_*others*_ distributions to assess the severity of FBA FC alterations; (3) pairwise comparisons of FBA *I*_*self*_ measures were conducted on network-specific *I*_*self*_ distributions based on seven resting-state functional networks; (4) FBA *I*_*self*_ was assessed for correlation with recorded HAE measurements.

### Pairwise comparisons of whole-brain FBA ***I***_***self***_ distributions

**Figure 3:**
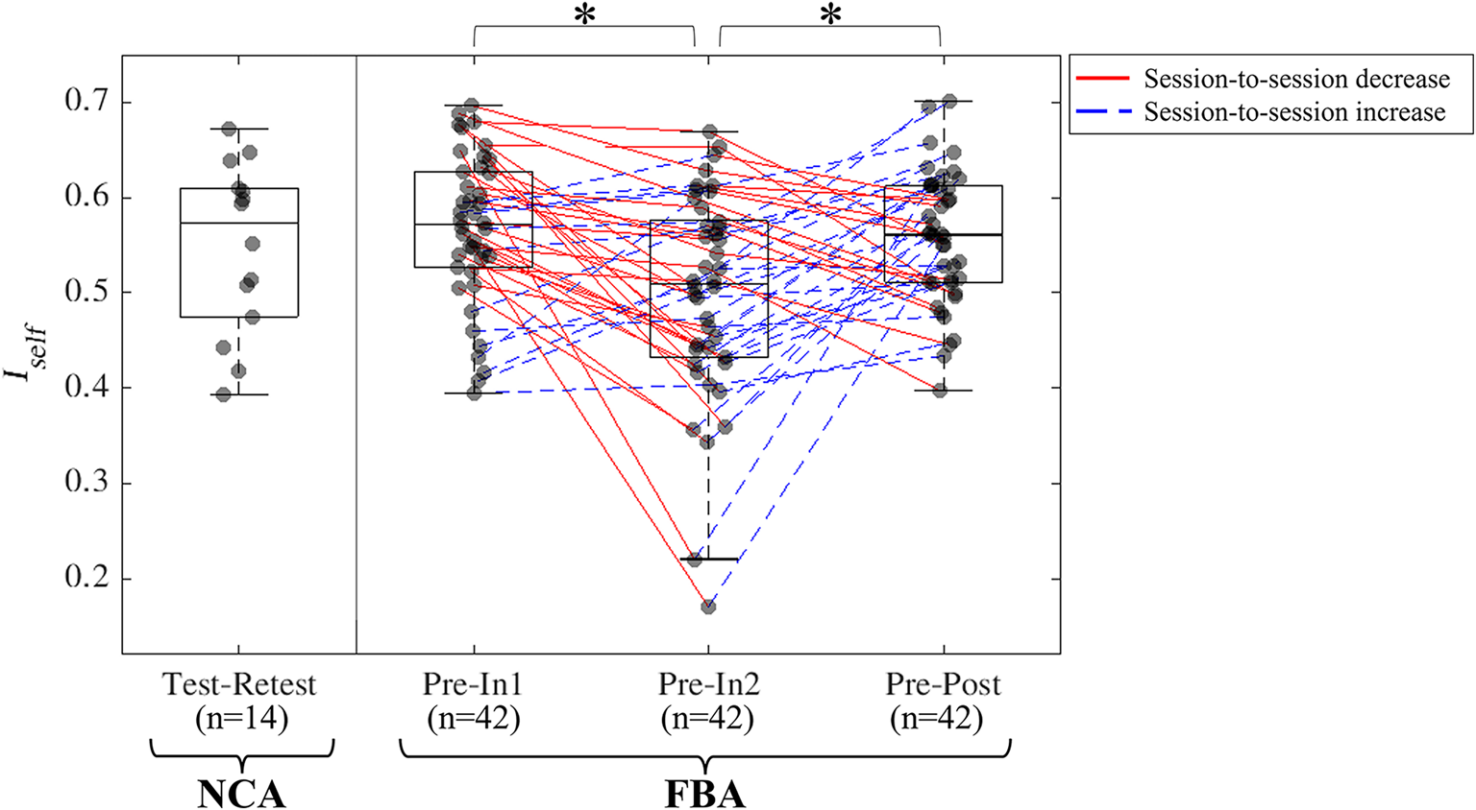
Box-and-whisker plots for each session-wise comparison, derived from 14 non-collision sports athletes (NCA, left) and 42 football athletes (FBA, right), demonstrate that self-similarity (*I*_*self*_) distributions for FBA computed at *Pre-In2* were significantly different from those observed for *Pre-In1* and *Pre-Post*, but not from the NCA *I*_*self*_ distribution. The *I*_*self*_ values for each scan session pair (as indicated for each column) was computed as the Pearson’s correlation between an individual’s functional connectomes for the given sessions, with each individual’s *I*_*self*_ plotted as a gray circle. For FBA, colored lines connect datapoints representing the same participant in different session comparisons; solid red lines indicate decreasing *I*_*self*_, and dashed blue lines indicate increasing *I*_*self*_. Asterisks indicate a statistical significance at the *p*_FDR_ < 0.05 level (Wilcoxon signed rank test).

*I*_*self*_ distribution comparisons were conducted to test whether functional connectivity changed significantly in FBA over the course of the season. Statistically significant differences (Wilcoxon signed rank test, *p*_*FDR*_ < 0.05) in *I*_*self*_ were found between *Pre-In1* and *Pre-In2* (*p*_*FDR*_ = 0.005), and also between *Pre-In2* and *Pre-Post* (*p*_*FDR*_ = 0.015) (Fig. 3). In particular, FC similarity with *Pre* was significantly lower at *In2* relative to *In1* and *Post.* This is reinforced by examination of the greater spread of *I*_*self*_ measures at this time point. (We note that for *Pre-In2* two football athletes, who did not have unusually high HAE accumulation relative to other football athletes, exhibited low *I*_*self*_, but exclusion of these participants did not affect the finding of statistical significance in the difference in *I*_*self*_ distributions.)

### Comparing FBA ***I***_***self***_ against NCA ***I***_***self***_ and ***I***_***others***_

**Figure 4:**
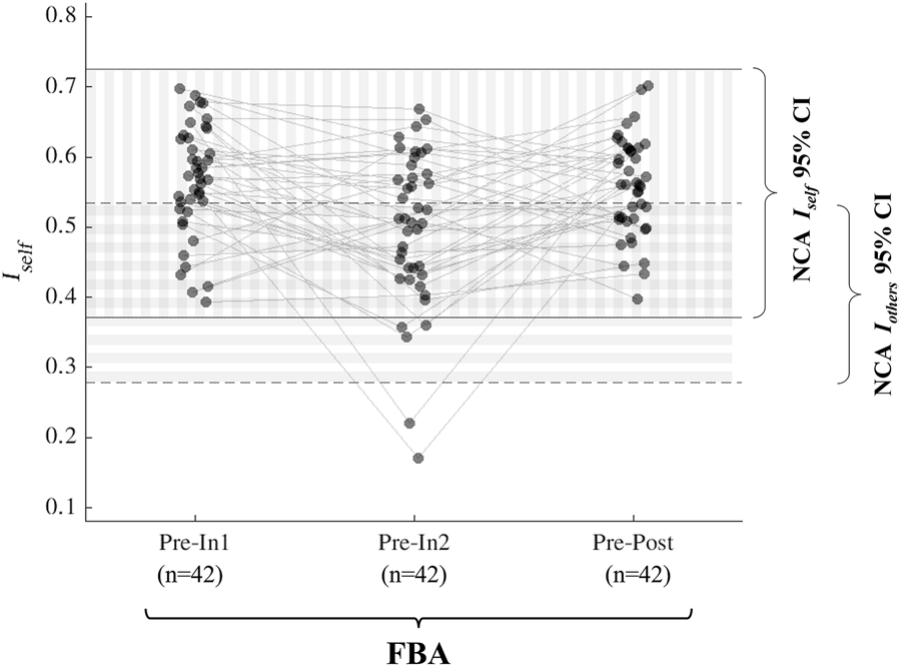
Values of *I*_*self*_ computed for football athletes (FBA) for *Pre-In1, Pre-In2,* and *Pre-Post* comparisons indicate that the FBA functional connectomes (FCs) were distributed in a manner more consistent with inter-individual comparisons at *Pre-In2*, as determined from examination of 95% confidence intervals (CIs) of non-collision athletes (NCA) *I*_*self*_ (vertically striped region, bounded by solid lines) and *I*_*others*_ (horizontally striped region, bounded by dashed lines). Datapoints plotted here represent the same points plotted in the right side of Fig. 3, now with the NCA *I*_*self*_ and *I*_*others*_ CIs superimposed. Gray lines connect datapoints from the same participant in the three different *I*_*self*_ distributions. The number of athletes falling within the 95% confidence interval of NCA *I*_*self*_ for *Pre-In1*, *Pre-In2*, and *Pre-Post* was 42, 37, and 42 out of 42 total FBA, respectively. The number of athletes falling below the upper limit the 95% confidence interval of NCA *I*_*others*_ for *Pre-In1*, *Pre-In2*, and *Pre-Post* was 11, 25, and 16 out of 42 total FBA, respectively.

Each of the FBA *I*_*self*_ distributions (*Pre-In1, Pre-In2*, and *Pre-Post*) were found to have no statistically significant differences in median when compared with NCA *I*_*self*_ (Wilcoxon rank sum test, *p*_*FDR*_ > 0.05 for all three tests). The number of FBA athletes for whom *I*_*self*_ fell within the 95% confidence interval, [0.37, 0.73], of the NCA *Test–Retest I*_*self*_ for each interval pairing was (*Pre-In1*) 42, (*Pre-In2*) 37, and (*Pre-Post*) 42 out of 42 (Fig. 4). The total number of FBA athletes for whom *I*_*self*_ fell below the upper limit (0.53) of the 95% confidence interval of NCA *I*_*others*_ for each interval pairing was (*Pre-In1*) 11*,* (*Pre-In2*) 25, and (*Pre-Post*) 16 out of 42. The Binomial test revealed that for *Pre-In2*, a significantly higher proportion (*p* = 0.022) of athletes had *I*_*self*_ falling within this *I*_*others*_ range, while no significance was found for *Pre-In1* and *Pre-Post*.

### ***I***_***self***_ analysis within resting state networks

**Figure 5:**
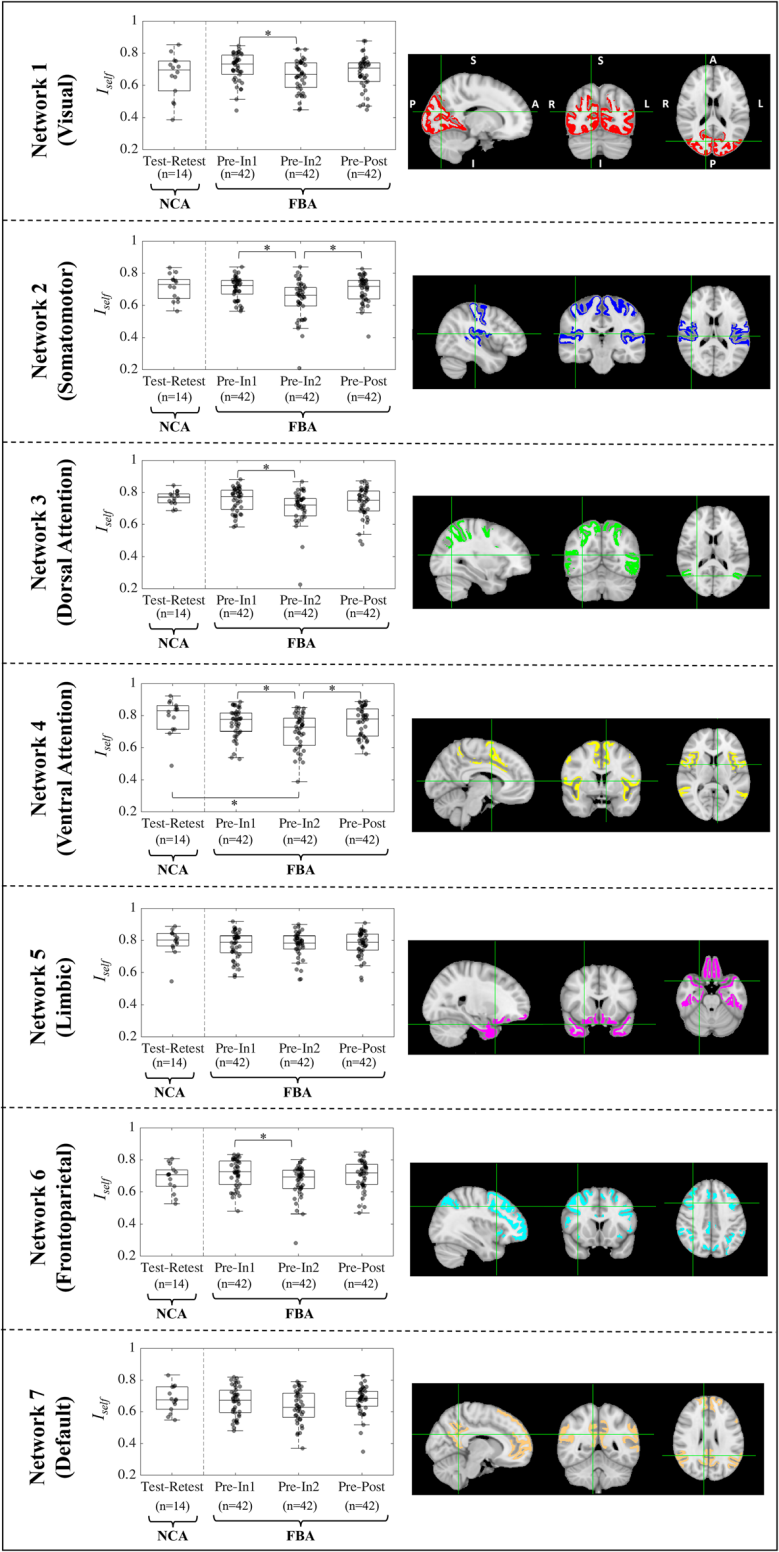
Network-specific analysis of *I*_*self*_ measures by population and session pairing indicate that football athletes (FBA) exhibit several statistically significant alterations across assessment sessions. (Left) Boxplots of *I*_*self*_ distributions for football athletes (FBA; *Pre-In1, Pre-In2*, and *Pre-Post*) and non-collision sports athletes (NCA; *Test–Retest*) within each of the seven Yeo functional networks^56^. Asterisks indicate statistical significance at the *p*_*FDR*_ < 0.05 level (Wilcoxon signed rank test for comparisons within FBA; Wilcoxon rank sum test for comparisons between NCA and FBA). (Right) Three-plane depiction of the primary gray matter extent of each of the Yeo networks. Green crosshairs indicate the location of brain slices shown for each network. Imaging directions are labeled on the first network (P = posterior, A = anterior, S = superior, I = inferior, R = right, L = left).

Network-specific *I*_*self*_ distributions were computed to determine the functional brain networks in which FBA functional connectivity changes were most prevalent. The trend observed in the whole-brain FC *I*_*self*_ comparisons, in which FC similarity with the *Pre* scan session was significantly different (Wilcoxon signed rank test, *p*_*FDR*_ < 0.05) at *In2* compared with *In1* and *Post*, was most profoundly seen in the somatomotor and ventral attention networks. Some single pairs of *I*_*self*_ distributions demonstrated significant differences in the remaining networks (Fig. 5), with the exception of the limbic and default mode networks. Of the pairwise tests between NCA *I*_*self*_ and FBA *I*_*self*_ distributions for each network, only the comparison of NCA *Test–Retest I*_*self*_ and FBA *Pre-In2 I*_*self*_ for the ventral attention network exhibited a statistically significant difference (Wilcoxon rank sum test, *p*_*FDR*_ < 0.05).

### Assessing the effect of accumulated HAEs on ***I***_***self***_

**Figure 6:**
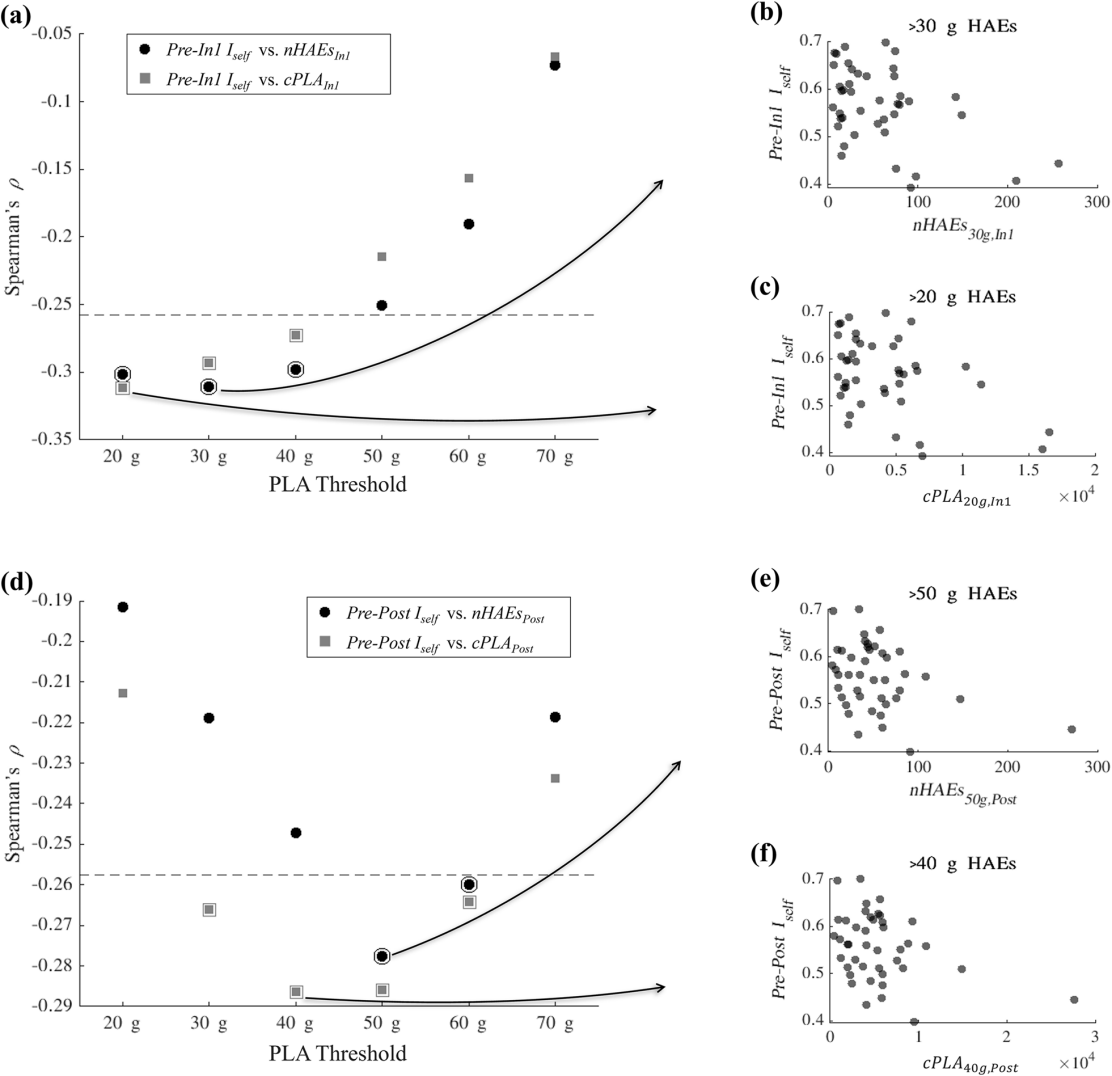
Correlation coefficients and selected scatterplots assessing relationship between *I*_*self*_ and head acceleration event (HAE) metrics. (**a**) Spearman’s correlation coefficient $\rho$ derived from comparison of *Pre-In1 I*_*self*_ with metrics measured from HAEs with peak linear acceleration (PLA) exceeding varying PLA thresholds. Specifically, *Pre-In1 I*_*self*_ was compared against the total number of HAEs experienced before *In1* (*nHAEs*_*In1*_; circle markers) and against the cumulative peak linear acceleration of HAEs experienced before *In1* (*cPLA*_*In1*_; square markers). The dashed gray line indicates the threshold below which Spearman’s $\rho$ was statistically significant (*p* < 0.05), derived from a one-tailed test assuming the null hypothesis $\rho \ge 0$ and alternative hypothesis $\rho <0$. Markers with an added outer circle or square indicate correlations for which a significant effect was also derived from bootstrap analysis of Spearman’s correlation (i.e., at least 95% of bootstrap resampling iterations produced Spearman’s $\rho <0$). (**b**) Scatterplot of *Pre-In1 I*_*self*_ versus *nHAEs*_*In1*_ exceeding 30 g (i.e., the threshold producing strongest Spearman’s $\rho$ when assessing *nHAEs*_*In1*_). (**c**) Scatterplot of *Pre-In1 I*_*self*_ versus *cPLA*_*In1*_ computed from HAEs exceeding 20 g (i.e., the threshold producing strongest Spearman’s $\rho$ when assessing *cPLA*_*In1*_). (**d**) Spearman’s correlation coefficient $\rho$ derived from comparison of *Pre-Post I*_*self*_ against *nHAEs*_*Post*_ (circle markers) and against *cPLA*_*Post*_ (square markers). The gray dashed line and markers with additional outer circle or square indicate significance as described in (a). (**e**) Scatterplot of *Pre-Post I*_*self*_ versus *nHAEs*_*Post*_ exceeding 50 g (i.e., the threshold producing strongest Spearman’s $\rho$ when assessing *nHAEs*_*Post*_). (**f**) Scatterplot of *Pre-Post I*_*self*_ versus *cPLA*_*Post*_ computed from HAEs exceeding 40 g (i.e., the threshold producing strongest Spearman’s $\rho$ when assessing *cPLA*_*Post*_). Note that no regression lines are shown in (**b**), (**c**), (**e**), and (**f**) because correlation was evaluated using the nonparametric Spearman’s rank correlation.

It is hypothesized that accumulation of HAEs leads to changes in functional brain connectivity, predicting a negative correlation between *I*_*self*_ and the number of HAEs experienced by a player. No significant correlations were found between *Pre-In2 I*_*self*_ and HAEs occurring before the *In2* scan session for any of the three HAE metrics tested at any PLA threshold (thus these data are not displayed). Spearman’s correlation coefficient $\rho$ and the associated *p*-values for comparisons of *Pre-In1 I*_*self*_ and *Pre-Post I*_*self*_ against the corresponding *nHAEs* and *cPLA* metrics are shown in Table 2. Significant negative correlations (*p* < 0.05; left-tailed Spearman’s) were observed when comparing *Pre-In1 I*_*self*_ with *nHAEs*_*In1*_, *Pre-In1 I*_*self*_ with *cPLA*_*In1*_, *Pre-Post I*_*self*_ with *nHAEs*_*Post*_, and *Pre-Post I*_*self*_ with *cPLA*_*Post*_ for varying PLA thresholds (see Table 2 and Fig. 6). Significance of negative correlations evaluated via bootstrapped analysis of the Spearman’s correlation (i.e., > 95% of bootstrap resampling iterations producing $\rho <0$), as documented in Fig. 6, further supported the observed correlations. *Pre-In1 I*_*self*_ and *Pre-Post I*_*self*_ were also tested for correlation with *aPLA* (average PLA of HAEs), but no significant correlation was found at any PLA threshold (these data are not displayed).

**Table 2:** Spearman’s correlation coefficient $\rho$ and associated *p*-values (based on left-tailed test), computed using HAEs above varying PLA thresholds, for comparisons of *Pre-In1* and *Pre-Post I*_*self*_ against *nHAEs* and *cPLA*.

*I*_*self*_ sessions	*PLA* threshold (g)	*I*_*self*_ versus *nHAEs*		*I*_*self*_ versus *cPLA*	
		Spearman’s $\rho$	*p*-value (left-tailed)	Spearman’s $\rho$	*p*-value (left-tailed)
*Pre-In1*	20 g	− 0.301	0.026*	− 0.312	0.023*
	30 g	− 0.311	0.023*	− 0.293	0.030*
	40 g	− 0.298	0.027*	− 0.272	0.041*
	50 g	− 0.251	0.055	− 0.215	0.086
	60 g	− 0.191	0.113	− 0.156	0.161
	70 g	− 0.073	0.322	− 0.067	0.337
*Pre-Post*	20 g	− 0.192	0.112	− 0.213	0.088
	30 g	− 0.219	0.082	− 0.266	0.044*
	40 g	− 0.247	0.057	− 0.286	0.033*
	50 g	− 0.278	0.038*	− 0.286	0.033*
	60 g	− 0.260	0.048*	− 0.264	0.045*
	70 g	− 0.219	0.082	− 0.234	0.068

No significant correlations were found for comparisons between *Pre-In2 I*_*self*_ and HAE metrics, so data for these analyses are not displayed. Asterisks (*) indicate a statistically significant Spearman’s correlation (left-tailed *p* < 0.05, uncorrected).

## Discussion

Variations in self-similarity (*I*_*self*_) of longitudinal resting-state fMRI functional connectivity in high school American football athletes exposed to head acceleration events (HAEs) were observed and found to correlate with HAE exposure over the course of a competitive season. Critical among these variations was (1) a decrease in within-individual *I*_*self*_ measures between baseline assessments and those obtained late in the competitive season, and (2) these within-individual *I*_*self*_ measures exhibited significantly increased overlap with inter-individual similarity measures obtained between non-identical individuals who had not been exposed to repetitive HAEs. This may be interpreted to suggest that continued exposure to repetitive HAEs could result in an individual’s FC no longer acting as a meaningful fingerprint that reflects the typical degree of *I*_*self*_^38^, with concomitant implications for their (near-term) neurological health and development. Short-term *I*_*self*_ was most strongly (negatively) correlated with essentially all events achieving a mild (roughly 20–30 g) or greater acceleration, while long-term *I*_*self*_ was most strongly (negatively) correlated with accumulation of events that exceeded a more severe (greater than 40–50 g) acceleration.

### FBA functional self-similarity declines late in play season

Relative to the pre-season scan session (*Pre*), deviation of whole-brain functional connectivity measures in FBA was strongest late in the competition season. *Pre-In2* whole-brain *I*_*self*_ was significantly lower than *Pre-In1* and *Pre-Post* for FBA, indicating that players’ own FC similarity (relative to the *Pre-*season scan session) decreased between the *In1* and *In2* scan sessions. This FC similarity was higher at the *Post* scan session as compared to *In2*, suggesting recovery of *Pre-*season functional connectivity patterns after the end of the play season. Many other studies support the notion of functional connectivity alterations occurring in collision sports athletes after collision activity seasons^1,11,32–34^.

A key finding of the present study is the observation of decreased within-individual similarity late in the competition season (at *In2*) as compared to pre-season, but not at the post-season scan (*Post*). Late-season brain changes in high school FBA have also been observed in previous studies using connectivity and MR spectroscopy in distinct cohorts. For example, Abbas et al.^1^ reported significant differences in the number of rs-fMRI default mode network connections (relative to pre-season) in FBA at in-season months 1 and 3, as well as 5 months post-season, but not at in-season months 2 and 4. Bari et al.^25^ reported significant changes in metabolite levels between *Pre-In2* and between *In2-Post,* reflecting a similar trend of late-season brain changes followed by post-season recovery*.*

### Degree of FBA within-individual self-similarity increased overlap with inter-individual similarity

The degree of functional connectivity changes in FBA represented at *In2* is severe enough that some athletes had *Pre-In2 I*_*self*_ measures that are closer to the similarity expected to be obtained from comparison of different individuals (*I*_*others*_) than to typical *I*_*self*_ values. While previous work has demonstrated significant functional connectivity changes as a result of participation in collision sports, the “severity” of such changes remains difficult to assess when athletes are asymptomatic. This study provides a potential means to quantify this severity through its comparison of participants’ FC *I*_*self*_ throughout the season with the degree of similarity of FCs observed between different individuals who were not exposed to HAEs.

Brain functional connectivity as observed through resting state fMRI has been studied as a means for identifying individuals from a group^36,38,57,58^, suggesting that individuals can be correctly identified to a great extent via functional connectivity patterns which act like a fingerprint. The distribution of NCA *I*_*others*_ is lower than that of NCA *I*_*self*_ (Fig. 4)*,* as *I*_*others*_ reflects the FC similarity between different individuals. At *In2*, a significantly higher proportion (compared to controls) of FBA have *I*_*self*_ within the range of NCA *I*_*others*_, with five athletes’ *I*_*self*_ so low that it falls outside the confidence interval of NCA *I*_*self*_. This indicates that the FC change between *Pre* and *In2* is substantial enough that some participants no longer exhibit their unique identifiable FC patterns. In the context of FC fingerprinting (i.e., identification of a subject based on their FC), this results in reduced subject identifiability, which may imply that these FC changes were more prominent than the lack of symptoms would suggest.

### Assessing correlation between ***I***_***self***_ and accumulated HAEs

As per the prior hypothesis, this study suggests that accumulation of HAEs leads to, among many documentable alterations in brain chemistry and function, both a near and long-term decrease in self-similarity of brain functional connectivity in football athletes. Given that the assessed NCA group is expected to be physically active but not to engage in (appreciable levels of) collision-based activity, the differences between the longitudinal behavior of FBA and NCA reasonably imply that HAEs are likely to be causal with regard to the changes here observed in *I*_*self*_. This inference is made stronger by the similarity of the contrast between FBA and NCA in previous studies involving independent cohorts of subjects, in which accumulation of deviation during HAE exposure was accompanied by recovery from that deviation with subsequent rest.

Analysis of correlations between *I*_*self*_ and the number of HAEs (*nHAEs*) experienced by players demonstrated a negative correlation between *nHAEs*_*In1*_ and *Pre-In1 I*_*self*_ as well as between *nHAEs*_*Post*_ and *Pre-Post I*_*self*_ values, suggesting that accumulation of HAEs is associated with a reduction in players’ *Pre*-season FC similarity both within the play season and after its conclusion. We note that the significance of the *p-*values associated with these correlations would not survive multiple comparisons correction, and thus should be interpreted cautiously. Correlations between functional connectivity changes and cumulative HAEs have also been demonstrated in other studies^9,33^, with these results being supportive of the finding of appreciable near-term reductions in seed-based connectivity by^1^.

Interestingly, our analysis did not demonstrate a correlation between *Pre-In2 I*_*self*_ values and the total number of HAEs experienced prior to *In2*. It has been suggested that changes in functional connectivity may stem from neurological adaptions made in response to a marked increase in HAEs (such as the start of the play season)^1^. Thus, one hypothesis explaining our study’s observed associations between *I*_*self*_ and HAE metrics is that functional connectivity changes may occur primarily following the onset and initial accumulation of HAEs, after which functional connectivity may remain relatively consistent in this newly “adaptive” state until the athlete is given sufficient recovery time free of any HAEs. This would suggest that by the time point of *In1*, only certain athletes had accumulated enough HAEs to result in FC changes (hence the correlation between HAEs and *Pre-In1 I*_*self*_), while by the time point of *In2*, nearly all athletes had exceeded the HAE accumulation necessary to induce FC changes relative to *Pre* (hence the overall decrease in *Pre-In2 I*_*self*_ yet lack of correlation with the further accumulation of HAEs). *Pre-Post I*_*self*_ appeared to recover to early-season levels, yet now exhibited a correlation with total HAEs when HAEs of a mild (e.g., 20–30 g) level were omitted. This suggests that while the FC profiles of many athletes recovered subsequent to cessation of HAE accumulation, those who had not yet recovered were more likely to have sustained a greater number of severe (i.e., higher PLA) HAEs throughout the season^59^.

### Toward a threshold for HAEs associated with physiological brain alterations

Currently, concerns regarding the precision of PLA measurements^39^ limit the determination of a precise PLA threshold of HAEs which lead to physiological brain alterations (and thus may be injurious). However, football accelerometer data is still especially useful for studying the relationship between cumulative HAEs with pathology related to neurological disorders—e.g., chronic traumatic encephalopathy^60^. A review of previous studies across multiple imaging modalities reveals patterns linking physiological brain alterations with cumulative exposure to both mild and severe HAEs in contact sports athletes. Specifically, cumulative exposure to more severe HAEs—i.e., those with PLA exceeding approximately 50 g, corresponding to approximately the 80th percentile of events exceeding 20 g^40^—has been found to be correlated with increased cerebrospinal fluid volumes^8^, neurometabolic alterations^25^, and cerebrovascular reactivity (CVR) changes^6^ in cohorts similar to the current study. Further, the current study found greater accumulation of such severe HAEs to be correlated with lower long-term *I*_*self*_. Cumulative exposure to comparatively mild HAEs (e.g., lower PLAs in the range 20–40 g) has been found to be correlated with changes in white matter (WM) fractional anisotropy (FA) measures^15^ and with near-term changes in whole brain functional connectivity in the present study. From these observations, it may be that the structural damage (e.g., that reflected in WM FA measures) resulting from the high number of mild HAEs leads to increased reliance on collateral processing mechanisms in the brain (reflected in changes in functional connectivity). We note that these mild HAEs (20–40 g PLA) represent approximately 70% of all HAEs with PLA above 20 g that are experienced by youth football athletes^40^. This hypothesis would then be consistent with the findings of Abbas et al.^1^, in which the most significant alterations in connectivity were associated with periods of time involving increases in HAE exposure, most of which is of a lesser severity. The observation that more severe HAE accumulation best correlates with other physiologic changes such as neurometabolic, CVR, and brain tissue alterations could indicate that these changes are in systems/structures that are more robust against mechanical insults (i.e., more resistant to HAEs with low PLA). Alternately, it may simply be that a longer period of time with HAE-induced physiologic changes must pass before sustained alteration of local metabolic activity can result in alterations in biochemical concentrations, and before changes in local glia are induced that would lead to altered CVR. Under this hypothesis, the correlation of such alterations with more severe HAEs would not necessarily indicate that more severe HAEs are the driving force of the observed changes. Rather, a higher accumulation of more severe HAEs would simply be a natural consequence of a greater period of time of sustained HAE exposure.

### Network-level functional connectivity changes associated with injury

The observed network-specific functional connectivity changes may suggest that repetitive exposure to HAEs can cause physiological brain changes similar to those observed following concussion, even in the absence of obvious symptoms or a concussion diagnosis. Analysis of FC similarity with the *Pre*-season scan session throughout the play season revealed that the somatomotor and ventral attention networks most profoundly demonstrated a decrease in FC similarity at *In2* followed by trends suggesting a recovery of *Pre-*season FC similarity at *Post*. Previous work has demonstrated a link between somatomotor functional changes and sports-related concussion. For instance, increases in relative homogeneity of functional connectivity in the sensorimotor cortex^29^ as well as reduction of functional connectivity in the somatomotor network^61^ have been shown to follow concussion. In addition, neurometabolic changes have been demonstrated within the motor cortex post-injury in concussed football athletes^62^ and due to HAEs^13,25,26^. Thus, the somatomotor network connectivity changes observed in our study may suggest that exposure to HAEs can cause physiological brain changes similar to those observed following concussion, despite not manifesting diagnosable symptoms. In addition, functional connectivity patterns have been shown to be altered in athletes sustaining anterior cruciate ligament (ACL) injuries^63,64^, demonstrating that neuroplastic changes can result even from injuries which are not directly applied to the central nervous system. Such observations of functional connectivity alterations in instances of clear injury raise concern for the health of athletes experiencing similar alterations in the absence of obvious symptoms. Given that repetitive concussion and HAE exposure may lead to long-term health risks^65^, these findings—consistent with the discussion above related to an injurious threshold—suggest that allowing recovery time between periods of HAE exposure may be necessary to prevent the accumulative effects of HAEs and promote the long-term health of football athletes.

Despite the current study’s lack of significant longitudinal *I*_*self*_ differences in the default mode network (DMN), it is of interest to note that the DMN has previously been shown to undergo changes in both asymptomatic football athletes exposed to HAEs^32,66^ and concussed athletes^30^. In the present study, comparisons of *Pre-In1* against *Pre-In2 I*_*self*_ and *Pre-In2* against *Pre-Post I*_*self*_ produced uncorrected *p*-values of 0.046 and 0.034, respectively, indicating the possibility of changes occurring within the DMN despite these values not surviving the FDR multiple comparisons correction.

### Collision-free time may allow for recovery

Given that results from this study suggest recovery of pre-season FC self-similarity between *In2* and *Post*, high school FBA may be capable of recovering from within-season functional connectivity changes after a sufficient period of time without (or possibly with substantially reduced) collision activities has passed. Athletes sustaining a full concussion may require up to 3–4 weeks for proper recovery^67^. It has also been shown that implementing prolonged waiting periods before returning to play after a concussion (with a median time of return to play of 12.2 days after injury) has resulted in decreased risk of repeat sports-related concussion^68^. Further, evidence has begun to grow supporting the idea that even athletes without diagnosed concussions require a prolonged period free of continued HAEs to fully recover from within-season alterations to brain physiology^8,12,25^.

We also wish to note that, in contrast to this study’s observation of recovered pre-season FC similarity at *Post*, some previous studies have reported functional connectivity changes between pre- and post-season scan sessions. One potential reason for this discrepancy is the time duration allowed after the season’s end before the post-season scan. Some other studies had post-season scan sessions which occurred within two weeks of the final game played^11,34^, while this study’s post-season scan was conducted 4–12 weeks after the season’s end. This suggests that differences from pre-season observed at post-season scans in other studies may be due to insufficient recovery time free of collision activities. In addition, the age of athletes studied (reported studies involve youth, high school, and collegiate players) may impact the degree and type of changes observed, as neural plasticity is age-dependent, and qualitatively different changes can occur at different ages in response to similar experiences^69^. It should also be noted that Abbas et al.^1^ reported significant hyperconnectivity in the default mode network in FBA compared with NCA, even at the pre-season time point, which suggests the hypothesis that lasting network changes result from years of participation in contact sports. This, along with the current study’s observation of negative correlation between *Pre-Post I*_*self*_ and full-season HAEs, would suggest that the post-season recovery observed in this study still may not represent full recovery of the athletes.

### Limitations

This study includes data from athletes attending three local high schools. A larger sample size covering more schools would include a greater diversity of athletes, better representing factors such as different socio-economic backgrounds, athlete health differences, and different play strategies. This larger sampling would help to generalize the results demonstrated here. In particular, a larger sample size of control athletes (NCA) would allow for stronger conclusions regarding the differences between NCA and FBA populations. For the present study, all available data of sufficiently high quality were incorporated.

It should also be noted that the repeatability of functional connectivity measures can be affected by multiple factors. Even in healthy populations not explicitly engaged in athletic programs or activities with high rates of repetitive HAEs, measures derived from resting state fMRI can be affected by factors such as mood^70^, arousal^71^, or caffeine intake^72^. Further, functional connectivity changes have been shown to be associated with participation in non-collision sports—such as distance running^73^ or badminton^74^—and more generally with participation in other motor-skills training programs^75,76^. Despite such factors, the tendency of FBA *Pre-In2 I*_*self*_ away from the NCA *I*_*self*_ distribution towards the NCA *I*_*others*_ distribution, coupled with the apparent recovery of FBA *Pre-*season functional connectivity several weeks after the halting of collision activities, suggests that the HAEs associated with football participation play a role in the decreased FC similarity shown at *In2*.

In addition, conclusions regarding association between FC alterations and cumulative HAEs are limited by several factors related to HAE monitoring, of which we focus here on three. First, though certain PLA thresholds did result in a significant correlation between *I*_*self*_ measurements and cumulative HAE metrics, the statistical significance of these correlations did not hold if a multiple comparisons correction was applied. We argue that the use of a multiple comparisons correction for the six different PLA thresholds would likely be too strict, as the HAE metrics based on different PLA thresholds are highly correlated with one another. Further investigation with a greater sample size would better confirm whether these correlations are truly present.

Second, the xPatch head acceleration monitoring devices provide estimates of peak linear acceleration having a 30% root-mean-square error^39^. This error represents added noise which may reduce the precision of thresholds when examining the correlation between *I*_*self*_ and accumulated HAEs. However, it is to be noted that the xPatch sensor was shown to produce lower levels of PLA measurement error than other commercially available sensor packages^39^, marking this sensor as one of the best available options, particularly when correctly placed on the mastoid process (as depicted by Lee et al.^40^).

Lastly, no video verification of HAEs was available for this study, meaning that precise numbers of recorded HAEs should be interpreted cautiously. While some studies do suggest video verification of head impacts should be used alongside head acceleration monitoring to prevent false-positive recordings of acceleration events^41,77,78^, we note that practical limitations (e.g., funding, manpower, and availability of video recordings) reduce the feasibility of implementation of video verification in all studies. Further, video verification is also unlikely to produce precise classification of HAEs. Limitations in video quantity or visibility of players can result in HAEs that are not captured on video^41^, or in other instances it can be unclear whether direct or indirect head impacts/accelerations occurred^77^. We argue that meaningful information can be gleaned from the HAE data used in the present study based on three justifications: (1) a 20 g PLA lower threshold is likely to exclude HAEs stemming from non-collision events such hard stops or cuts (that are not likely to be injurious)^42^; (2) the aggregated number of reported events is highly consistent across head-mounted devices^39^; and (3) given that the PLA error induced by the xPatch sensor is stochastic in nature, then analysis over an extended period of total counts of HAEs exceeding the 20 g threshold should provide an accurate picture of the aggregated exposure^10^.

## Conclusion

This study demonstrated that high school American football athletes undergo changes in brain functional connectivity over the course of a play season, though none sustained a clinically diagnosed concussion. Functional connectome self-similarity (*I*_*self*_) with individuals’ *Pre*-season session was shown to significantly decrease by the time of the second half of the play season (*In2*), while this self-similarity with the *Pre*-season FC was recovered by the *Post* scan session. The lower levels of *within-individual* self-similarity in the second half of the play season were found to be significantly overlapping with the distribution of *inter-individual* similarity among different individuals. This overlap at *In2* suggested that some athletes could be identified (e.g., as in fingerprinting) as different individuals when compared to their *Pre*-season FC. At a network level, these functional connectivity alterations were found to occur most prominently in the somatomotor and ventral attention networks. Both early and post-season connectivity changes were found to be negatively associated with HAE metrics. These results highlight that repetitive exposure to HAEs can result in significant changes in brain functional connectivity without diagnosable symptoms and suggest that recovery time free from continued exposure to acceleration events may be beneficial for the recovery of youth athletes’ brain physiology.

## Data Availability

The datasets analyzed during the current study are available from the corresponding author on reasonable request.
